# Binary reversals: a diagnostic sign in primary progressive aphasia

**DOI:** 10.1136/jnnp-2023-331662

**Published:** 2023-12-09

**Authors:** Eoin Mulroy, Lucy B Core, Anthipa Chokesuwattanaskul, Jeremy CS Johnson, Phillip D Fletcher, Charles R Marshall, Anna Volkmer, Jonathan D Rohrer, Chris JD Hardy, Martin N Rossor, Jason D Warren

**Affiliations:** 1 Dementia Research Centre, Department of Neurodegenerative Disease, UCL Queen Square Institute of Neurology, University College London, London, UK; 2 Division of Neurology, Department of Internal Medicine, King Chulalongkorn Memorial Hospital, Thai Red Cross Society, Bangkok, Thailand; 3 Cognitive Clinical and Computational Neuroscience Research Unit, Faculty of Medicine, Chulalongkorn University, Bangkok, Thailand; 4 Department of Neurology, St George's University Hospitals NHS Foundation Trust, London, UK; 5 Preventive Neurology Unit, Wolfson Institute of Population Health, Queen Mary University of London, London, UK; 6 Division of Psychology and Language Sciences, University College London, London, UK

**Keywords:** PRIMARY PROGRESSIVE APHASIA, FRONTOTEMPORAL DEMENTIA, DEMENTIA

## Abstract

**Background:**

Binary reversals (exemplified by ‘yes’/‘no’ confusions) have been described in patients with primary progressive aphasia (PPA) but their diagnostic value and phenotypic correlates have not been defined.

**Methods:**

We conducted a retrospective cohort study analysing demographic, clinical, neuropsychological, linguistic and behavioural data from patients representing all major PPA syndromes (non-fluent/agrammatic variant, nfvPPA; logopenic variant, lvPPA; semantic variant, svPPA) and behavioural variant frontotemporal dementia (bvFTD). The prevalence of binary reversals and behavioural abnormalities, illness duration, parkinsonian features and neuropsychological test scores were compared between neurodegenerative syndromes, and the diagnostic predictive value of binary reversals was assessed using logistic regression.

**Results:**

Data were obtained for 83 patients (21 nfvPPA, 13 lvPPA, 22 svPPA, 27 bvFTD). Binary reversals occurred in all patients with nfvPPA, but significantly less frequently and later in lvPPA (54%), svPPA (9%) and bvFTD (44%). Patients with bvFTD with binary reversals had significantly more severe language (but not general executive or behavioural) deficits than those without reversals. Controlling for potentially confounding variables, binary reversals strongly predicted a diagnosis of nfvPPA over other syndromes.

**Conclusions:**

Binary reversals are a sensitive (though not specific) neurolinguistic feature of nfvPPA, and should suggest this diagnosis if present as a prominent early symptom.

## Introduction

Diagnosis of primary progressive aphasia (PPA) is challenging even for expert clinicians.^1^ Given the current dearth of objective biomarkers in these diseases, clinical phenotyping remains paramount. ‘Binary reversals’—selection of the wrong alternative from a pair of candidate opposite verbal responses (most often ‘yes’/‘no’), frequently with spontaneous self-correction—have been reported as a phenomenon marring everyday communication in people with PPA.^2–5^ This symptom may constitute a specifically neurolinguistic feature, rather than reflecting a more generalised deficit of behaviour regulation.^5^ However, the diagnostic value of binary reversals has not been clarified.

Here we addressed this issue in a large, well characterised patient cohort representing all canonical syndromes of PPA and the behavioural variant of frontotemporal dementia (bvFTD). We assessed the prevalence of binary reversals in relation to syndromic diagnosis and associated clinical, neuropsychological and behavioural features. Based on clinical experience and previously published observations,^2–5^ we hypothesised that binary reversals would be more prevalent in the non-fluent/agrammatic variant of PPA (nfvPPA) than other syndromes and would be associated with linguistic deficits rather than behavioural abnormalities.

## Methods

### Assessment of patients

We assessed all patients in our active research cohort at the Dementia Research Centre who fulfilled consensus diagnostic criteria for nfvPPA, logopenic variant PPA (lvPPA), semantic variant PPA (svPPA) or bvFTD.^6 7^ All had syndromes of mild-to-moderate severity and supportive brain MRI with minimal cerebrovascular burden. Patient group characteristics are summarised in table 1.

**Table 1:** Clinical, cognitive and behavioural characteristics of patient subgroups with/without binary reversals

Characteristic	nfvPPA^*^	bvFTD			lvPPA		svPPA	
Binary reversals	Present	Absent	Present	P value	Absent	Present	Absent	Present
General								
No. of patients (%)	21 (100)	15 (56)	12 (44)	–	6 (46)	7 (54)	20 (91)	2 (9)
Sex (male:female)	13:8	11:4	9:3	0.92	5:1	6:1	13:7	0:2
Handedness (right:left)	20:1	14:1	12:0	NT	6:0	6:1	19:1	2:0
Age at testing (years)	71.5 (8.1)	70.0 (7.8)	62.5 (5.1)	*<0.01*	66.1 (8.9)	68.7 (8.9)	65.8 (7.1)	69.3 (9.2)
Duration (years)(med (IQR)^†^	3.0 (2.4)	4.1 (1.3)	5.8 (2.5)	*0.03*	4.3 (1.7)	6.0 (1.8)	4.9 (1.8)	7.3 (3.0)
MMSE	**20.5** (**9.5**)	25.1 (5.2)	**21.3** (**5.8**)	0.08	**21.0** (**5.4**)	**11.1** (**8.6**)	**22.9** (**7.4**)	**12.5** (**7.8**)
Neuropsychology								
*Executive functions*								
TMT A (/150 s)	**81.6** (**49.4**)	**65.1** (**42.8**)	**83.5** (**53.6**)	0.34	**102** (**56.8**)	**99.3** (**41.2**)	**48.5** (**27.9**)	**117** (**46.7**)
TMT B (/300 s)	**209.9** (**91.3**)	**171.0** (**87.0**)	**219.9** (**97.6**)	0.19	**268.5** (**55.4**)	**278.3** (**53.1**)	**128.3** (**87.5**)	**253.0** (**66.5**)
Phonological fluency	**5.2 (6**)	**9.5** (**4.9**)	**5.2 (5**)	*0.04*	**4.2** (**3.6**)	**3.4** (**5.2**)	**8.2** (**5.5**)	**2.5** (**3.5**)
Category fluency	**8.8** (**6.9**)	**11.5** (**7.6**)	**8.7** (**6.8**)	0.33	**5.3** (**3.7**)	**5.3** (**7.9**)	**5.8** (**4.2**)	**4.5** (**6.4**)
Stroop: colour (/90 s)	**85.4** (**27.1**)	**43.6** (**21.2**)	**59.5** (**23.1**)	0.08	**78.8** (**22.5**)	**79.0** (**18.9**)	**53.6** (**21.8**)	**66.0** (**33.9**)
Stroop: word (/90 s)	**71.6** (**18.4**)	**28.5 (186**)	**39.8** (**21.7**)	0.03	**49.0** (**22.4**)	**52.3** (**8.1**)	**32.8** (**21.3**)	**64.5** (**36.1**)
Stroop: ink (/180 s)	**154.4 (40**)	**97.1** (**52.3**)	**122.1** (**55.1**)	0.25	**166.3** (**33.5**)	**169.3** (**28.4**)	**96.5** (**47.4**)	**139.0** (**58.0**)
Verbal working memory								
Digit span forward (/12)	**3.95** (**2.5**)	8.6 (2.4)	6.8 (2.6)	0.09	**4.3** (**2.3**)	**1.7** (**1.5**)	8.3 (2.1)	6.5 (0.7)
Digit span reverse (/12)	**2.3** (**2.0**)	5.9 (2.7)	**2.7** (**2.7**)	*0.046*	**2.5** (**1.2**)	**1.9** (**2.3**)	6.8 (2.8)	3.5 (4.95)
Language functions								
GNT (/30)	**13.2** (**7.6**)	16.0 (10.3)	**12.5** (**9.9**)	0.30	**11.7** (**7.5**)	**2.3** (**4.4**)	**1.2** (**3.8**)	**0.0** (**0.0**)
BPVS (/150)	**122.3** (**41.5**)	137.8 (19.9)	**100.8** (**55.1**)	*0.04*	140.0 (11.0)	**83.0** (**59.9**)	**76.5** (**46.8**)	**31.0** (**41.0**)
NART (/50)	14.4 (13.2)	36.2 (8.1)	23.5 (12.7)	*<0.01*	30.0 (9.7)	10.4 (10.7)	19.7 (13.3)	**0.0 (N/A^‡^**)
Word repetition (/45)	**32.2** (**13.8**)	NT	NT	–	**41.8** (**3.7**)	**24.9** (**16.2**)	44.1 (1.2)	**37.5** (**2.1**)
Sentence repetition (/10)	**3.2** (**2.6**)	NT	NT	–	**5.2** (**2.3**)	**2.4** (**1.6**)	**7.5** (**2.3**)	**4.5** (**2.1**)
Sentence construction§ (/25)	**15.3** (**10.9**)	NT	NT	–	**18.8** (**4.9**)	**6.7** (**8.9**)	**17.8** (**8.3**)	22.0 (‡)
PALPA55 (/24)	**16.9** (**5.6**)	NT	NT	–	**18.8** (**2.9**)	**10.9** (**4.3**)	**20.3** (**5.7**)	**13.5** (**2.1**)
Baxter Spelling Test (/30)	15.1 (8.8)	NT	NT	–	15.2 (5.7)	**3.1** (**6.1**)	12.5 (7.6)	**6.0 (N/A^‡^**)
Episodic memory								
RMT faces (/50)	**34.2** (**7.6**)	**32.9** (**8.2**)	**31.8** (**7.1**)	0.72	**35.5** (**8.0**)	**28.1** (**5.0**)	**32.9** (**5.7**)	**27.0** (**5.7**)
RMT words (/50)	**39.0** (**9.3**)	**36.9** (**9.9**)	**36.2** (**8.5**)	0.84	**35.8** (**11.0**)	**29.3** (**7.5**)	**33.4** (**7.1**)	**34.0** (**9.9**)
Other skills								
GDA (/24)	4.7 (5.8)	11.2 (7.8)	6.5 (5.6)	0.10	**1.8** (**2.1**)	**0.7** (**1.5**)	11.3 (7.5)	**2.0** (**2.8**)
VOSP (/20)	14.9 (3.9)	14.8 (4.6)	**13.2** (**5.9**)	0.45	15.5 (3.2)	**12.3** (**4.1**)	16.8 (2.9)	**10.0** (**7.1**)
Behavioural changes								
Disinhibition (n (%)	5 (24)	13 (87)	11 (92)	0.68	0 (0)	3 (43)	12 (60)	2 (100)
Apathy (n (%))	11 (52)	13 (87)	10 (83)	0.81	1 (17)	5 (71)	8 (40)	2 (100)
Obsessiveness (n (%))	6 (29)	12 (80)	9 (75)	0.76	1 (17)	2 (29)	12 (60)	2 (100)
Aberrant motor (n (%))	7 (33)	11 (73)	8 (67)	0.71	0 (0)	3 (43)	5 (25)	1 (50)
Parkinsonism								
Present (n (%))	13 (62)^¶^	1 (7)	2 (17)	0.41	3 (50)	3 (43)	0 (0)	0 (0)

The table summarises demographic, clinical, behavioural and neuropsychological data for all participant groups, subdivided on the basis of whether or not they made binary reversals. Mean (SD) data are shown unless otherwise indicated; maximum scores on neuropsychological tests are shown in parentheses. Neuropsychological scores in bold indicate performance below the 10th percentile according to published norms or local normative data from the Dementia Research Centre research cohort of older healthy controls (n=40, 21 males, 19 females, mean age 68.0 (6.1)). bvFTD subgroups with and without binary reversals have been compared statistically, as case numbers in this diagnostic group made the comparison meaningful; significant differences between subgroups (p<0.05) are coded in italics. Not all neuropsychological tests were completed by all patients; numbers in the bvFTD group missing data for each test are presented in online supplemental table S2 in online supplemental material.

*All patients in the nfvPPA group exhibited binary reversals; two patients in this group fulfilled criteria for primary progressive apraxia of speech (ie, presentation with ‘pure’ speech apraxia and normal performance on key language tests: GNT, BPVS, PALPA55 and sentence construction).

†Estimated duration of symptoms.

‡Missing data left only one patient so SD could not be calculated.

§In-house written sentence construction task to assess output grammar.

¶Eight patients in the nfvPPA group had features of PSP or CBS; parkinsonism lacked diagnostic features in other syndromic groups.

BPVS, British Picture Vocabulary Scale; bvFTD, patient group with behavioural variant frontotemporal dementia; GDA, Graded Difficulty Arithmetic Test; GNT, Graded Naming Test; lvPPA, patient group with logopenic variant primary progressive aphasia; med, median; MMSE, Mini-Mental State Examination score; NART, National Adult Reading Test; nfvPPA, patient group with non-fluent/agrammatic variant of primary progressive aphasia; NT, not tested; PALPA55, Psycholinguistic Assessment of Language Processing in Aphasia sentence–picture matching subtest; RMT, Recognition Memory Test; svPPA, patient group with semantic variant primary progressive aphasia; TMT, Trail Making Test Parts A / B; VOSP, Visual Object and Space Perception Battery.

10.1136/jnnp-2023-331662.supp1 Supplementary data

Using a structured clinical survey, we recorded the presence (or absence) of binary reversals and other potentially relevant behavioural symptoms following illness onset (online supplemental table S1) in online supplemental file 1), consulting with each patient’s primary caregiver or equivalent close informant; informants were invited to provide examples of the symptom. Patients underwent neurological examination and a comprehensive neuropsychological assessment (table 1). In addition, we recorded whether binary reversals were associated with parkinsonism and/or a diagnosis of corticobasal syndrome (CBS) or progressive supranuclear palsy (PSP).

### Statistical analyses

Statistical analyses were performed using SPSS V.28.0 and R (V.4.3.1). Kolmogorov-Smirnov tests and Levene’s tests were first conducted to check for normality and homogeneity of variance, respectively. A one-way Analysis of Variance (ANOVA) assessed for age differences and a Kruskal-Wallis test for differences in Mini Mental State Examination (MMSE) scores between patient groups (irrespective of binary reversal status). The Kruskal-Wallis test was used to compare illness duration in the nfvPPA cohort versus patients with and without binary reversals in other syndromic categories. For binarised data (symptoms present/absent), group differences were assessed using χ^2^ tests. Neuropsychological and behavioural associations of binary reversals were assessed within the bvFTD group (the largest diagnostic group) by comparing patient subgroups with and without binary reversals using independent-samples t-tests or Mann-Whitney U tests; results from two-tailed tests are reported. A binomial logistic regression model was used to assess the likelihood of having a diagnosis of nfvPPA versus other syndromes in the presence of binary reversals, incorporating age, symptom duration and MMSE score as covariates in the model and also without these covariates. Alpha threshold 0.05 was used for all comparisons. Multiple comparison correction was not performed, given the relatively small sample size (substantial risk of failing to detect a true effect) and lack of independence of surveyed characteristics.

## Results

Data from 83 patients (21 nfvPPA, 13 lvPPA, 22 svPPA, 27 bvFTD) were available for analysis (table 1). Within the nfvPPA group, two patients fulfilled criteria for primary progressive apraxia of speech^8^ (table 1). Patient groups did not differ significantly in age (*F*(3,79) = 2.08, p=0.11) or overall disease severity indexed using MMSE (χ^2^(3) = 7.12, p=0.07).

Binary reversals were reported across syndromic groups, although with widely varying prevalence: binary reversals were reported in all patients with nfvPPA, but significantly less frequently (p<0.05) in patients with lvPPA (54%), bvFTD (44%) and svPPA (9%). Informant descriptions indicated that reversals most commonly involved mis-selection of ‘yes/no’, but diverse other examples were produced (including ‘left/right’, ‘he/she’, ‘up/down’, ‘open/shut’, ‘good/bad’, ‘hot/cold’, ‘north/south’); non-verbal communication gestures (thumbs up/down, head nod/shake) could also be affected. The subgroups of patients with bvFTD and lvPPA who reported binary reversals had significantly longer mean symptom duration than the nfvPPA group (both p<0.05), whereas symptom duration in patients with bvFTD, lvPPA and svPPA who had no binary reversals was similar to the nfvPPA group (p>0.05).

**Figure 1:**
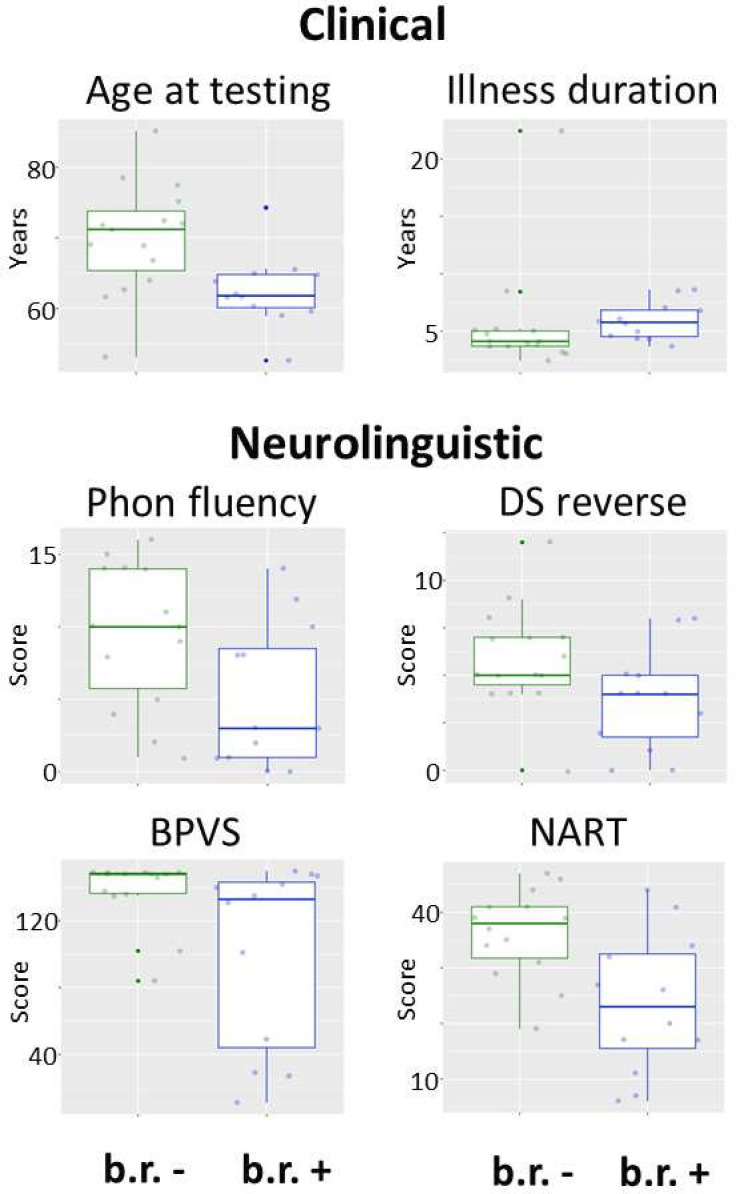
Significant phenotypic associations of binary reversals in the behavioural variant frontotemporal dementia group. The figure shows box-and-whisker plots of clinical and neurolinguistic characteristics significantly associated with the presence of binary reversals, across individual patients with behavioural variant frontotemporal dementia (the patient group in which associations could be most reliably assessed; see text) at the prescribed threshold (p<0.05) (non-significant associations are plotted in online supplemental figure S1; see also text and online supplemental table S2). Boxes represent the IQR, and whiskers indicate the overall range of values in each group; the horizontal line in each box represents the median; in each panel, data for patients who exhibited binary reversals at the time of assessment (b.r. +) are presented on the right (in blue) and data for patients who did not exhibit reversals (b.r. −) on the left (in green). Binary reversals were significantly associated with younger age at assessment, longer symptom duration and more severe deficits of phonological fluency, phonological working memory, single word comprehension and reading. BPVS, British Picture Vocabulary Scale (a measure of single word comprehensions); DS reverse, reverse digit span (a measure of phonological working memory); NART, National Adult Reading Test; Phon fluency, phonological fluency (number of words generated to a target initial letter in 1 minute).

Within the bvFTD group (table 1, figure 1), binary reversals were significantly associated with younger age at testing (t(25) = 2.85, p<0.01), longer illness duration (Mann-Whitney U=46.00, p=0.03) and more severe deficits of phonological fluency (t(24) = 2.22, p=0.04), phonological working memory (reverse digit span) (t(25) = 2.10, p=0.046), single word comprehension (Mann-Whitney U=43.50, p=0.04) and reading (National Adult Reading Test, t(24) = 3.10, p<0.01; Stroop word reading, Mann-Whitney U=126.5, p=0.03). Reversals were not significantly associated with other executive, general cognitive or behavioural deficits in the bvFTD group (all test statistics presented in full in online supplemental table S2 and online supplemental figure S1). A qualitatively similar pattern of more severe language deficits in patients exhibiting binary reversals was present in the lvPPA and svPPA groups (table 1).

Parkinsonian features were present in 62% of patients with nfvPPA (half with a diagnosis of PSP or CBS) but were less prevalent in other syndromic groups and not consistently associated with binary reversals (table 1).

After covarying for potentially confounding factors of age, illness duration and overall severity (and applying Firth’s bias reduction method^9^ to account for the universality of binary reversals in the nfvPPA group), the presence of binary reversals conferred significantly higher ORs for a diagnosis of nfvPPA versus all other syndromes (OR=5.07, 95% CI (2.74 to 10.04), p<0.001) versus all other PPA syndromes (OR=5.07, 95% CI (2.74 to 10.01), p<0.001) and versus individual syndromes of bvFTD (OR=5.30, 95% CI (2.46 to 10.73), p<0.001), lvPPA (OR=3.87, 95% CI (1.41 to 8.84), p<0.001) and svPPA (OR=5.39, 95% CI (2.94 to 11.25), p<0.001). Similarly significant results were obtained from the model using only binary reversals as the independent predictor (online supplemental table S3).

## Discussion

We have shown that binary reversals strongly predict a diagnosis of nfvPPA, developing in a high proportion (here 100%) of patients with this syndrome and significantly more frequently than in other PPA syndromes or bvFTD. Binary reversals were uncommon in svPPA and though encountered in around half of patients with lvPPA and bvFTD, developed later and/or in the context of more severe cognitive impairment in these syndromes than in nfvPPA. This feature may therefore have higher diagnostic specificity earlier in the course of the illness.

While this study does not elucidate the pathophysiological mechanism, it is noteworthy that, within the bvFTD group, patients with binary reversals performed significantly less well on neurolinguistic measures (phonological fluency, phonological working memory, word comprehension and reading) than patients who did not make reversals, whereas the two subgroups had otherwise comparable executive, general cognitive and behavioural profiles. This suggests that the development of binary reversals can form part of the neurolinguistic phenotype of bvFTD.^10^ Although it was not possible to analyse the specific associations of binary reversals in the nfvPPA group (since reversals were universal in this group), no single behavioural feature nor the presence of clinical parkinsonism, PSP or CBS was required for binary reversals to manifest. Taken together, our findings suggest that binary reversals are a neurolinguistic phenomenon, rather than a non-specific consequence of executive dysregulation, in line with previous reports of similar reversals in aphasic stroke.^11^ On the other hand, impaired response inhibition (as indexed by impaired Stroop task performance) was present in all patient groups exhibiting binary reversals, which may signify a complex interplay of causative and permissive factors.^2 3^

Further work is needed to characterise the semiology of binary reversals and the circumstances that provoke them. Here we simply recorded the occurrence of the symptom; quantifying the frequency and severity of binary reversals and tracking their longitudinal development in the individual patient would give a more nuanced picture and establish how this symptom relates to other features of the illness. Anecdotally, a similar phenomenon occurs in nfvPPA patients speaking languages other than English: this requires substantiation. The neural basis of the symptom also remains to be defined. Our nfvPPA cohort is fairly typical neuropsychologically and neurologically of other published series,^12 13^ and (considered alongside previous observations^2 3^) the propensity of this syndrome to manifest binary reversals may reflect the targeting of fronto-subcortical circuitry by causative tauopathies. However, unless binary reversals have led to an important communication failure, this symptom may not be volunteered.^4^ We propose that clinicians suspecting PPA should seek a history of binary reversals and—particularly where early and prominent—this curious phenomenon may constitute a useful diagnostic clue. Recognition will enable investigation and management, including speech and language therapy for patients in whom binary reversals present a significant issue for communication in daily life.
