# Supplementary material for: Binary reversals: a diagnostic sign in primary progressive aphasia
Source: J Neurol Neurosurg Psychiatry. 2023 Dec 9;95(5):477–80. doi: 10.1136/jnnp-2023-331662 (PMC11041576; doi:10.1136/jnnp-2023-331662)
Supplement: Supplementary data [file jnnp-2023-331662supp001.pdf]

Supplementary Material. Binary reversals: a diagnostic sign in primary progressive aphasia, by Eoin Mulroy et al

Table S1. Clinical survey of binary reversals and other behavioural symptoms

Symptom	Explanation	Yes	No	Please give examples:
**Binary reversals**	Tends to reverse opposites (e.g. Yes/No, Up/Down, etc) when speaking or otherwise communicating (e.g. head nod / shake, etc)			
**Disinhibition**	Socially inappropriate behaviour, lack of adherence to social norms, loss of manners or decorum			
**Apathy**	Loss of interest, drive and motivation, decreased initiation of activities			
**Obsessiveness**	Activities or ideas which s/he engages in or expresses obsessively, e.g. repetitive or ritualistic routines that s/he seems compelled to perform			
**Aberrant motor**	Paces without purpose, repeatedly dresses or undresses, excessively fidgety			

The survey was completed by each patient's primary caregiver (or equivalent close informant), following an initial explanation by the researcher. For this first study we simply recorded whether or not the symptom had definitely been noted since the onset of the illness. For each item the caregiver was invited to give examples from the patient's daily life.

Mulroy E, *et al. J Neurol Neurosurg Psychiatry* 2024; 95:477–480. doi: 10.1136/jnnp-2023-331662

Table S2. Complete test statistics and sample sizes comparing phenotypic associations in bvFTD patients with and without binary reversals

Characteristic	Absent: n[a]	Present: n[a]	Test Statistic	Degrees of freedom	P value
**General**					
Sex	15	12	X^2 = 0.01 (Fisher exact sig. = 1)	1	0.92
**Age at assessment (years)**	**15**	**12**	**t = 2.85**	**25**	**<0.01**
Symptom duration (years)	15	12	U = 46.00	NA	0.03
MMSE	15	12	t = 1.83	25	0.08
**Neuropsychology**					
Executive functions					
TMT A	14	12	t = -0.97	24	0.34
TMT B	14	12	t = -1.35	24	0.19
**Phonological fluency**	**14**	**12**	**t = 2.22**	**24**	**0.04**
Category fluency	14	12	t = 0.99	24	0.33
Stroop: Colour	14	12	t = -1.82	24	0.08
**Stroop: Word**	**14**	**12**	**U = 126.50**	**NA**	**0.03**
Stroop: Ink	14	12	t = -1.18	24	0.25
Working memory					
Digit span forward	15	12	U = 55.50	NA	0.09
**Digit span reverse**	**15**	**12**	**t = 2.10**	**25**	**0.046**
Language functions					
GNT	15	12	U = 68.50	NA	0.30
**BPVS**	**14**	**12**	**U = 43.50**	**NA**	**0.04**
**NART**	**14**	**12**	**t = 3.10**	**24**	**<0.01**
Episodic memory					
RMT Faces	15	12	t = 0.37	25	0.72
RMT Words	14	11	t = 0.20	23	0.84
Other skills					
GDA	13	11	t = 1.70	22	0.10
VOSP	13	12	t = 0.76	23	0.45
**Behavioural changes**					
Disinhibition	15	12	X^2 = 0.17 (Fisher exact sig. = 1)	1	0.68
Apathy	15	12	X^2 = 0.06 (Fisher exact sig. = 1)	1	0.81
Obsessiveness	15	12	X^2 = 0.10 (Fisher exact sig. = 1)	1	0.76
Aberrant motor	15	12	X^2 = 0.14 (Fisher exact sig. = 1)	1	0.71
**Parkinsonism**					
Present	15	12	X^2 = 0.68 (Fisher exact sig. =0.57)	1	0.41

The Table presents results for all comparisons of clinical, neuropsychological and behavioural characteristics between subgroups of behavioural variant frontotemporal dementia (bvFTD) patients with (Present) and without (Absent) binary reversals. Between-group differences were assessed as follows. Mann-Whitney U tests were used to compare Stroop Word, Digit Span forward, GNT, and BPVS scores, due to violations of the normality assumption. Chi^2 and Fisher's exact tests were used to assess presence of behavioural changes and parkinsonism. Independent sample t-tests were used for all other measures. Significant comparisons ($p < 0.05$) are in bold. [a]Some patients did not

complete all tests. BPVS, British Picture Vocabulary Scale; GDA, Graded Difficulty Arithmetic Test; GNT, Graded Naming Test; MMSE, Mini-Mental State Examination score; n, number; NA, not applicable; NART, National Adult Reading Test; RMT, Recognition Memory Test; TMT, Trail Making Test Parts A / B; VOSP, Visual Object and Space Perception Battery.

Table S3. Results of binomial logistic regression analysis on the diagnostic predictive value of binary reversals, with and without correcting for covariates.

Comparison	OR	95% CI	p-value
Including covariates			
nfvPPA vs all other syndromes	5.07	2.74, 10.04	<0.001
nfvPPA vs other PPA	5.07	2.74, 10.01	<0.001
nfvPPA vs lvPPA	3.87	1.41, 8.84	<0.001
nfvPPA vs svPPA	5.39	2.94, 11.25	<0.001
nfvPPA vs bvFTD	5.30	2.46, 10.73	<0.001
Without covariates			
nfvPPA vs all other syndromes	4.42	2.35, 9.28	<0.001
nfvPPA vs other PPA	4.79	2.64, 9.67	<0.001
nfvPPA vs lvPPA	3.62	1.30, 8.53	<0.001
nfvPPA vs svPPA	5.87	3.47, 10.83	<0.001
nfvPPA vs bvFTD	3.98	1.82, 8.86	<0.001

This analysis examined the effect of binary reversals on the likelihood of having a diagnosis of nfvPPA versus other syndromes, with (above) and without (below) adjusting for age, symptom duration and MMSE score (see main text). Firth's bias reduction method was applied. bvFTD = behavioural variant frontotemporal dementia; CI = confidence interval; lvPPA = logopenic variant PPA; nfvPPA= non-fluent/agrammatic variant PPA; OR = odds ratio; PPA = primary progressive aphasia; svPPA = semantic variant PPA.

Figure S1. Plots of non-significant phenotypic associations of binary reversals in the behavioural variant frontotemporal dementia group

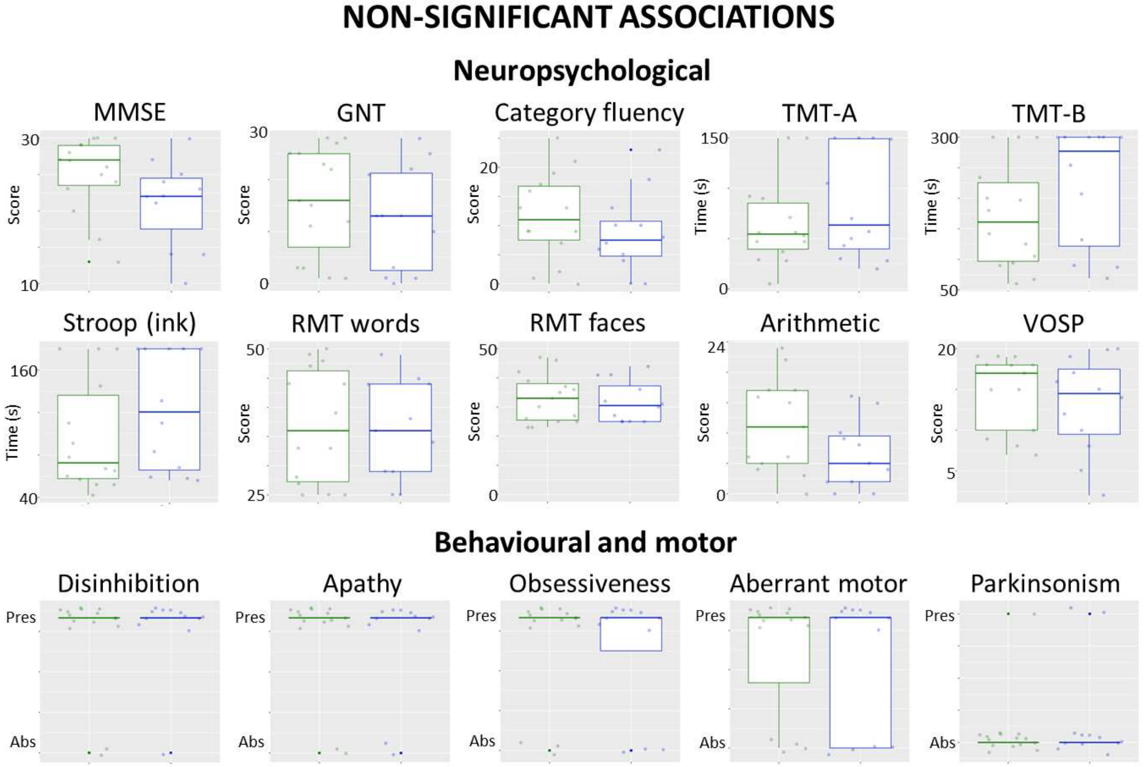

This Figure is a companion to Figure 1. It shows box-and-whisker plots of other associations (clinical, neuropsychological and behavioural characteristics) assessed in comparisons of patients with and without binary reversals, across individual patients with behavioural variant frontotemporal dementia (the patient group in which associations could be most reliably assessed; see main text). Associations shown here were all non-significant at the prescribed threshold ($p > 0.05$) (significant associations are plotted in Figure 1; see also Table S2). Boxes represent the interquartile range, and whiskers indicate the overall range of values in each group; the horizontal line in each box represents the median; in each panel, data for parients who exhibited binary reversals at the time of assessment are presented on the right (in blue) and data for parients who did not exhibit reversals on the left (in green). **GNT**, Graded Naming Test; **MMSE**, Mini-Mental State Examination score; **RMT**, Recognition Memory Test; s, seconds; **TMT**, Trail Making Test Parts A / B; **VOSP**, Visual Object and Space Perception Battery.

Mulroy E, *et al. J Neurol Neurosurg Psychiatry* 2024; 95:477–480. doi: 10.1136/jnnp-2023-331662
